# Tumor-Associated CSF MicroRNAs for the Prediction and Evaluation of CNS Malignancies

**DOI:** 10.3390/ijms161226150

**Published:** 2015-12-07

**Authors:** Tarek Shalaby, Michael A. Grotzer

**Affiliations:** Department of Oncology, University Children’s Hospital, CH-8032 Zurich, Switzerland; tarek.shalaby@kispi.uzh.ch

**Keywords:** cerebrospinal fluid, CNS cancers, microRNA, biomarkers

## Abstract

Cerebrospinal fluid (CSF) is a readily reachable body fluid that is reflective of the underlying pathological state of the central nervous system (CNS). Hence it has been targeted for biomarker discovery for a variety of neurological disorders. CSF is also the major route for seeding metastases of CNS malignancies and its analysis could be informative for diagnosis and risk stratification of brain cancers. Recently, modern high-throughput, microRNAs (miRNAs) measuring technology has enabled sensitive detection of distinct miRNAs that are bio-chemicallystable in the CSF and can distinguish between different types of CNS cancers. Owing to the fact that a CSF specimen can be obtained with relative ease, analysis of CSF miRNAs could be a promising contribution to clinical practice. In this review, we examine the current scientific knowledge on tumor associated CSF miRNAs that could guide diagnosis of different brain cancer types, or could be helpful in predicting disease progression and therapy response. Finally, we highlight their potential applications clinically as biomarkers and discuss limitations.

## 1. Background

**Figure 1:**
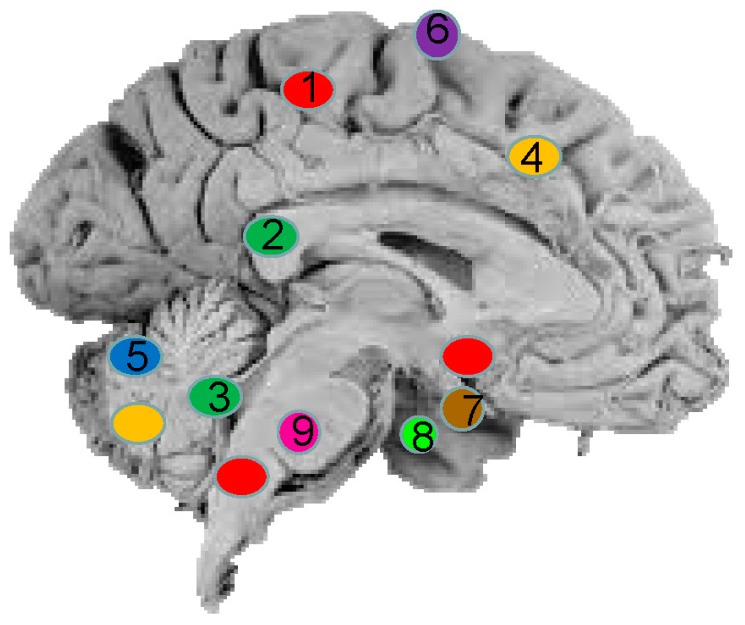
Main types and locations of primary brain tumors: (1) Gliomas, (2) Supratentorial Ependymoma, (3) Infratentorial Ependymoma, (4) Astrocytomas, (5) Medulloblastomas, (6) Meningioma, (7) Craniopharyngioma, (8) Pituitary tumors, and (9) Schwamannomas.

The most frequently occurring brain malignancies in adults are metastatic brain cancers that have spread to the central nervous system (CNS) from other body parts, followed by glioblastomas [1,2] while the most common brain tumors in children are astrocytomas, medulloblastomas and ependymomas [3] (Figure 1). The current diagnostic tools for brain cancer, including clinical manifestations, neuroimaging Magnetic resonance imaging (MRI) and computed tomography (CT) and histology, although indispensable, have limitations and are used relatively late in the pathogenesis. Clinical signs and symptoms are often subtle and unspecific while sensitivity and specificity of neuroimaging differ widely with the type of cancer and location [4]. Histology remains the gold standard analysis that is most frequently used for achieving diagnosis, yet tumor biopsy is an invasive method, which is associated with the risk of brain hemorrhage and neurologic damage. In addition some brain-lesions are not surgically accessible and accordingly are not amenable to biopsy. Hence it becomes a clinical imperative to define biological markers that are sensitive enough to aid in the detection of brain malignancy, preferably at an early stage.

Several blood tumor markers are presently used for a wide range of cancer types including cancer antigen (CA)-125, α-fetoprotein (AFP), CA15-3/CA27.29 and prostate-specific antigen (PSA) for ovarian, liver, breast and prostate cancer, respectively, as well as β-2-microglobulin (B2M) for chronic lymphocytic leukemia, β-human chorionic gonadotropin (β-hCG) for testicular cancer; CA19-9 for gastric, gall bladder and pancreatic cancer; urokinase plasminogen activator (uPA) and plasminogen activator inhibitor (PAI-1) for breast cancer; and thyroglobulin for thyroid cancer [4,5].

However, one of the challenges that contributes to the paucity of biomarkers in the serum of CNS malignancies is the blood–brain barrier, which is thought to prevent the release of tumor-specific molecules into the circulation. The Cerebrospinal fluid (CSF) has thus been investigated in the search for brain tumor markers.

Profiling of circulating miRNA expression in the CSF has linked specific miRNAs to CNS malignancies. They can distinguish between different types of brain cancers, reflect disease activity and could be associated with drug resistance [1,2,6,7]. Since deregulated miRNA expression is an early event in tumorigenesis, their presence in CSF may represent a gold mine of biomarkers for early brain cancer detection, which can contribute greatly to treatment success [8,9,10]. Hence great efforts have been made in conducting research evaluating their diagnostic value in CNS cancers, reviewed in [10].

In this review, we provide a short overview on the diagnostic significance of miRNA circulating in CSF of patients with brain cancer disease and discuss limitations and challenges.

## 2. miRNAs Circulating in Body Fluids

**Figure 2:**
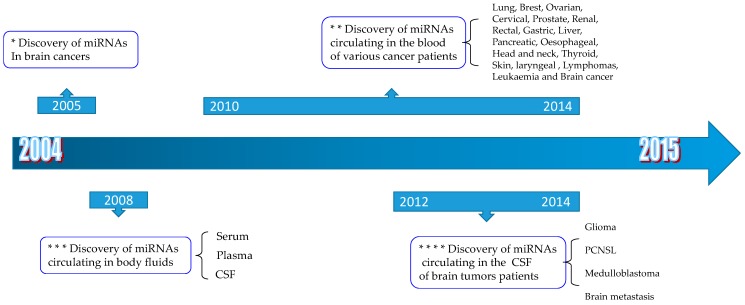
Time line depicting the discoveries of body fluids’ miRNAs and their contribution to cancer detection. CSF: Cerebrospinal fluid. * [36,37,38]; ** Lung [39], Brest [40], Ovarian [41], Cervical [42], Prostate [43], Renal [44], Rectal [45], Gastric [46], Liver [47], Pancreatic [48], Oesophageal [49], Head and neck [50], Thyroid [51], Skin [52], laryngeal [53], Lymphomas [54], Leukaemia [55] and Brain cancer [56,57,58,59]; *** Serum [11,31], Plasma [14], CSF [12]; **** Glioma [2,60,61], PCNSL [29,61,62], Medulloblastoma [22,61], Brain metastasis [2,61].

While miRNAs are found mainly inside cells, a considerable number of miRNAs have recently been discovered circulating in a stable, extracellular form—in various body fluids, including plasma, serum, CSF, urine, and saliva [11,12,13,14,15] (Figure 2). However, the origin and the function of these circulating miRNAs (c-miRNAs) are not well understood. Cancer cells in culture have also been reported to export miRNAs into the extracellular environment [16,17,18,19]. There have been relatively few published reports, including ours, on the isolation of miRNAs from cell-conditioned media [17,20,21,22]. However, the function of such secreted miRNAs remains essentially unclear. Because of their stability and easy detection in body fluids, an increasing number of studies have focused on c-miRNAs potential as non-invasive biomarkers and as therapeutic targets or tools for cancers. Several reports have described that deregulated c-miRNAs in body fluids are closely associated with the clinical course of various brain lesions including cancer [23,24,25,26,27,28,29,30]. For example, but not limited to, Lawrie *et al.* [31] reported that serum level of miR-21 is reversely associated with relapse-free survival in patients with diffuse large B-cell lymphoma. While the levels of miR-486, miR-30d, miR-1, and miR-499 were significantly associated with overall survival of non-small-cell lung cancer [32]. In another publication, it was reported that levels of miR-141 in serum could distinguish between patients with prostate cancer and healthy subjects [14]. Ng *et al.* [33] showed that miR-92 can detect colon cancer in plasma samples. Moreover, Yamamoto *et al.* [34] reported that patients with hepatocellular carcinoma have high serum levels of miR-500, which were significantly reduced after surgery and returned to normal levels. While Wong *et al.* [35] showed that plasma level of miR-184 is much higher in cancer patients with early and advanced squamous cell carcinoma of the tongue than in normal individuals and is reduced in patients after the surgical removal of the primary tumor reviewed in [16]. The significant differences between c-miRNA expression profiles in body fluids of cancer patients and those of healthy individuals and importantly the association of c-miRNAs with the clinical course of the disease has raised the possibility that they may serve as a novel minimally invasive and sensitive approach to detect cancer in its early stages [2,24,25].

## 3. Cerebrospinal Fluid (CSF) as Diagnostic Window for the Pathological State of the Central Nervous System (CNS)

### 3.1. CSF Characteristics

CSF is formed mainly in the ventricular choroid plexus and distributed within the ventricular system and subarachnoid space. It contains 15 to 45 mg/dL protein, 50–80 mg/dL glucose and 0–5 mononuclear white blood cells/mL and serves as a medium for the nutrients delivery to the CNS as well as carrying away waste. Because CSF bathes the CNS and comes into close contact with any pathological brain tissue, it is considered an ideal source of molecules related to brain cancers as it can be accessed readily for diagnosis as well as for longitudinal disease monitoring during therapy. Approximately up to 20 mL of CSF is usually collected for testing through a lumbar puncture (spinal tap) where a needle is inserted, usually between the 3rd and 4th lumbar vertebrae. Such a laboratory smear poses only minimal risk to the patient. In a routine clinical setting, CSF analysis is widely used for diagnostic and prognostic purposes, and it provides critically important information for a number of neurological diseases [63,64].

### 3.2. CSF Analysis for Brain Cancer Markers Detection

Cancer cells infiltrate the CSF either directly from primary CNS cancers through direct spread from tumors that are in close contact with the CSF or through blood brain barrier penetration from cancers that have spread to the CNS from other body parts [2]. CSF cytology, in which CSF is examined under a microscope to look for neoplastic cells (presence or absence) is currently considered the gold standard test for diagnosis, tumor staging, and therapy decisions in many types of cancer such as medulloblastoma, lymphoma, and metastatic carcinomas [65]. Although indispensable, this method bears neither quantification nor molecular analysis of tumor cells, lacks sensitivity and assignment to a particular tumor is often not possible [65,66,67].

To maximize the chances of finding brain cancer markers in the CSF, it has been realized that it is necessary to detect changes at the molecular level rather than waiting for a macroscopic tumor to emerge [65]. Various methods and new technologies are being tested for *in vitro* assessment of CSF looking for potential markers for CNS cancers including: proteochemical and immunophenotypic studies by flow cytometry (that provides information about cell surface protein expression), molecular genetic analyses of CSF [67], immunocytochemistry, immunoglobulin heavy chain (IgH) rearrangement which analyzes the clonality of the antibodies being produced, polymerase chain reaction, fluorescence *in-situ* hybridization (FISH), DNA single cell cytometry, capillary electrophoresis and mass spectrometry reviewed in [4,65]. Proteomic profiling of CNS malignancies has revealed that free immunoglobulin light chains and antithrombin III, a serine protease inhibitor that is associated with neo-vascularization, are differentially expressed in some brain cancers [68,69]. Levels of lactate dehydrogenase (LDH4 and LDH5), cell surface proteins such as β2-microglobulin, CXC chemokines (CXCL13 and CXCL12), soluble CD27, vascular endothelial growth factor, urokinase and tissue plasminogen activator have been evaluated in the CSF as brain cancer markers [70,71,72,73,74], Table 1. However none of these markers have been clinically validated [64]. On the other hand levels of conventional tumor markers (which are used to monitor treatment and detect recurrence in serum of patients with various malignancies) such as cancer antigen 125 (CA125), CA19-9, CA72-4, CA715-3, alpha-fetoprotein (AFP), Neuron-specific Enolase (NSE), cytokeratin-19 fragment (CYFRA 21-1), Epidermal Growth Factor Receptor (EGFR) and beta- human chorionic gonadotropin (β-hCG) β-human chorionic gonadotropin were tested in the CSF of brain cancer patients. Elevated levels of such markers in the CSF but not in serum were found to be relatively specific for some brain cancer [70,71,72,73,74,75]. Then again, sensitivities and specificities have varied widely, reviewed in [4]. Loss of 1p and 19q and mutant IDH1 (Isocitrate Dehydrogenase 1) as well as O6-methylguanin-DNA-methyltransferase (MGMT) promoter methylation were detected in glioma patients, and efforts to locate these aberrations in circulating tumor cells are ongoing [76,77]. Abnormality in CSF component has in addition been reported in brain cancer cases. These abnormalities include increased leukocytes (>4/mm^3^), elevated protein (>50 mg/dL), and decreased glucose (<60 mg/dL). Although indicative of brain cancer these CSF abnormalities are nonspecific [29,78,79,80]. However, in general, the use of the above-mentioned markers has been limited due to their poor sensitivity and specificity. Moreover, there was no clear correlation with the type of cancer or response to treatment observed reviewed in [4].

**Table 1:** Markers in body fluids that have potential application for brain cancer detection.

Marker	Sample	Type of Brain Cancer	References
* AT III	* CSF	* CNS lymphoma	[68,81,82]
EGFR	CSF	Brain metastases from lung adenocarcinoma	[83]
Pro-inflammatory (* IL-1β, IL-6, IL-8, IL-12, * GM-and * TNF-α) and anti-inflammatory cytokines (IL-4, IL-10), and (* VEGF, * bFGF)	Blood Serum	Glioblastoma	[84]
* CYFRA 21-1, * NSE and * CEA	CSF	Meningeal carcinomas	[85]
* CXCL13 plus interleukin 10	CSF	CNS lymphoma	[74]
* VEGF receptor 1 and 2	CSF	Leukemia CNS metastasis	[86]
miR-21 and miR-15b	CSF	Glioblastoma	[25]
miR-19, miR-21, and miR-92a	CSF	* PCNSL	[25]
miR-10b and miR-21	CSF	Glioblastoma and brain Metastasis	[2]
Members of miR-200 family	CSF	Brain metastases from lung and breast cancers	[2]
miR 210	Serum	Gliomas	[81]
miRNA-205	Serum	Glioma	[87]
miR-21	CSF	Glioma	[60]
MiR-451, -711, -223 and -125b	CSF	Glioblastoma, medulloblastoma, brain metastasis and lymphoma	[61]
MiR-935	CSF	Only brain metastasis	[61]
* GFAP and * EGFR	Serum	Gliomas	[88]
Interleukin-10	CSF	PCNSL	[89]
* PGD2	CSF	Medulloblastoma	[90]
* IgG levels	CSF	Cerebral low-grade lymphoma	[91]
CXCL13 and CXCL12	CSF	CNS lymphoma	[92]
* MIC-1/* GDF15	CSF	Glioblastoma	[93]
Vascular endothelial growth factor (VEGF) and stromal cell derived factor (SDF)-1	CSF	Brain metastases from lung and breast cancers	[94]
β-2 microglobulin	CSF	Myeloma of the central nervous system	[94]
Apolipoprotein A-II	CSF	Pediatric brain tumors (medulloblastoma , high-grade glioma, atypical rhabdoid tumor, astrocytoma, plexus carcinoma and anaplastic ependymoma, germ cell tumor)	[95]
VEGF	CSF	Leptomeningeal metastasis	[96]
VEGF and serologic (recoverin)	CSF and serum	Malignant glioma	[97]
Mitochondrial DNA mutations	CSF	Medulloblastoma	[98]
c-kit	CSF	Germ cell tumors	[99]
Human chorionic gonadotropin (hCG) and α-fetoprotein (AFP)	Serum and CSF	Intracranial germ cell tumors	[100]
Prostaglandin D synthase (β-trace)	CSF	Meningeal hemangiopericytoma	[101,102]
CD27	CSF	CNS lymphoma	[103]
β-hCG	CSF and serum	Brain metastases from gestational trophoblastic tumors	[104]

* Abbreviations: Antithrombin III (A III), Cerebrospinal fluid (CSF), central nervous system (CNS), Interleukin-1 beta (IL-1β), granulocyte macrophage-colony stimulating factor (GM-CSF), tumor necrosis factor-alpha (TNF-α), vascular endothelial growth factor (VEGF), basic fibroblast growth factor (bFGF), cytokeratin-19 fragment (Cyfra 21-1), Neuron-specific Enolase (NSE), carcinoembryonic antigen (CEA), chemoattractant, chemokine (C-X-C motif) ligand 13 (CXCL13), vascular endothelial growth factor (VEGF), Primary central nervous system lymphoma (PCNSL), Glial fibrillary acidic protein (GFAP), Epidermal growth factor receptor (EGFR), Prostaglandin-D2 synthase (PGD2), Immunoglobulin G (IgG), Macrophage inhibitory cytokine-1 (MIC-1), Growth differentiation factor 15 (GDF15).

## 4. miRNAs as Potential Novel Candidates for Brain Cancer Markers in the CSF

Studying miRNAs as biomarkers in brain cancer tissue samples has created noticeable advances in cancer research. However, a key downside of the tissue-based approach is the need for surgical procedures in sample collection. Therefore, brain cancer biomarkers’ researchers have now turned their attention to the analysis of miRNAs present in biological fluids including CSF, which can be collected with minimal invasiveness and permit following the disease over time [4]. It has been speculated that c-miRNAs detected in the CSF of brain cancer patients—that originate from brain tumor cells—can be pro-tumorigenic [105]. They can transfer their oncogenic activity to recipient target cells where they change their properties by influencing molecular events of cancer-related processes such as growth, invasion and drug resistance. This exchange of miRNAs between primary tumors and target cells is an interesting and novel dimension to the regulation of a cell phenotype [106] and may be exceptionally important in malignancies that have a tendency for dissemination. It is possible that primary brain tumors with intra cranial dissemination property or tumors that metastasize to the brain from distance cancers may release miRNAs in the CSF with clinically relevant oncogenic signatures. Hence studying miRNAs circulating in CSF of brain tumor patients may be used to decode the molecular features of the underlying malignancy as well as their metastasis [10].

## 5. Diagnostic and Prognostic Value of CSF miRNAs in Brain Cancers

Diagnosis of CNS involvement in neoplastic diseases, including both primary and metastatic cancers, is a major clinical intractability [4]. To date, no reliable biomarkers for the detection and risk stratification of CNS cancer have been identified. Great efforts have been made during the past decades in conducting research evaluating the diagnostic value of miRNAs in the CSF of patients with CNS cancers. Using qRT-PCR, Baraniskin *et al.* [25,62] investigated the diagnostic value of miRNAs that are expressed with significant levels in CSF samples from patients with gliomas. The authors found that out of the six miRNAs (miR-21, miR-15b, miR-196b, miR-92a and miR-204) studied miR-21 and miR-15b have the potential to function as novel biomarkers for the detection of gliomas. Both miR-21 and miR-15b were specifically expressed in CSF samples from patients with gliomas compared to control subjects Impressively, analysis of miR-15b and miR-21 in combined expression increased diagnostic accuracy with 100% specificity and discriminated between glioma patients and controls including those with carcinomatous brain metastases and primary CNS lymphoma [25,62].

In a study by Teplyuk and colleagues [2], the authors examined miRNAs in the CSF of patients with metastatic brain cancers, patients with glioblastoma multiforme (GBM), and non-neoplastic controls. They reported that profiling miRNAs in CSF of these patients allowed the discrimination between glioblastoma and metastatic brain tumors and reflects disease activity. Combined analysis of a group of seven cancer related miRNAs, using q-RT-PCR, enabled discrimination between GBM and metastatic brain cancers with more than 90% accuracy, suggesting that the presence of specific c-miRNAs circulating in the CSF of brain tumor patients could be indicators of cancer presence. miRNA-21 and miR-10b expression levels were significantly increased only in brain tumor lesions (in patients with GBM or brain metastases) compared to non-neoplastic conditions, while members of the miR-200 family were found solely in CSF of patients with brain metastases hence they could be used to discriminate between glioblastoma and metastatic brain tumors, an important consideration for cancer treatment. Furthermore, they showed that disease burden and treatment response can be monitored over several months by longitudinal miRNA profiles of miR-10b, miR-21 and miR-200 in the CSF of brain tumor patients [2]. Given that GBM is the deadliest glioma with a median survival of only 14 months despite the recent advances in intensive therapeutic strategies [58] and that approximately one-third of cancer patients will develop brain metastases, more efforts were applied to study whether specific CSF miRNA profile can be generated to evaluate gliomas prognosis. In this setting, in a recent publication Shi and colleagues examined CSF from seventy patients with recurrent glioma for the levels of cancer-related miRNAs, and evaluated the values for prognosis. The authors reported that the CSF miR-21 circulating in the CSF levels is a promising indicator for glioma diagnosis and prognosis, and can predict tumor recurrence or metastasis. Their results particularly demonstrated that miRNA-21 expression in the CSF is associated with poor prognosis and tumor recurrence of glioma patients [60].

Primary central nervous system lymphoma (PCNSL) is another highly aggressive tumor that can lead to quick death if not diagnosed in time. The diagnosis of PCNSL can present a diagnostic challenge. It relies on histopathology of brain biopsies to the same extent as most brain tumors, while non-invasive tests to detect early tumor legions with sufficient diagnostic accuracy are not available yet. In an earlier work, Baraniskin *et al.* demonstrated the presence of notably stable miRNAs in the CSF of PCNSL patients. The group identified specific miRNAs that are expressed with significant levels in the CSF of patients with PCNSL compared to control. Using candidate approach and miRNA quantification by qRT-PCR, the group discovered that miR-19, miR-21, and miR-92a in CSF accurately discriminates patients with primary central nervous system lymphoma from other neurologic disorders controls, indicating significant diagnostic value and increased diagnostic accuracy with 95.7% sensitivity and 96.7% specificity [25,62,67]. On the same theme, Scott *et al.* conducted a review of the literature on CNS lymphoma diagnosis (1966 to October 2011) and extracted data regarding the usefulness of CSF cytology in the diagnosis of CNS lymphoma. The authors reported low sensitivity for CSF cytology (2%–32%), which is increased when combined with flow cytometry. Studying β2-microglobulin, and immunoglobulin heavy chain rearrangement as well as lactate dehydrogenase isozyme 5 in the CSF has improved CSF cytology sensitivity but not specificity. Interestingly, miRNA analysis has more than 95% specificity in the diagnosis of CNS lymphoma [29].

Medulloblastoma (MB) is the most common malignant brain tumor in children. It includes various subtypes with group 3 and 4 subtypes being clinically distinct with regard to metastasis and prognosis, which may also manifest in a difference in their miRNA spectra. The presence and biological role of ex-miRNAs in MBs was unknown until our lab recently examined the existence of ex-miRNAs in MB extracellular environment and showed that the MB cells release miRNAs in their spent culture medium. We used microarray analysis to unveil the identity and level of expression of key miRNAs excreted in culture-medium of three cell lines representing different MB subtypes, D341 and D283 (metastasis-related group 3 and group 4 MB subtypes) [107] and DAOY (sonic hedgehog-related). More than one thousand secreted miRNAs were identified in the culture medium in each of the MB cell lines tested. Among them a panel of miRNAs was specific to the culture medium of metastasis-related cell lines which represents the aggressive group 3 and 4 MB subtypes. 60 miRNAs were overexpressed and 52 underrepresented compared to DAOY culture medium. Interestingly, three metastasis-associated miRNAs (miR-1290, miR-125a, and miR-125b) were over-represented in culture-medium of metastasis-related MB cell lines and found to be significantly enriched in the CSF of the MB patient [22]. Although more samples are required to fully verify these results, our work presented the first evidence for the presence of miRNAs excreted extracellularly by MB cells and raises the possibility that investigations using larger sets of MB samples could lead in the near future to the discovery of CSF-derived miRNA markers, with diagnostic and prognostic significance and hopefully also with therapeutic potential.

In an elegant work by Wei and colleagues [108], the authors conducted systematic meta-analysis searching different electronic databases and sources for relevant articles on the topic “the diagnostic value of miRNAs for CNS cancers”. In this meta-analysis, a total of 299 CNS cancer patients and 418 controls were analyzed within 23 studies. Thirteen out of the 23 studies investigated miRNAs for the detection of glioma and 10 studies for PCNSL diagnosis. In all studies, levels of miRNAs expression were analyzed by qRT-PCR in patients CSF and blood in order to compare blood *versus* CSF based miRNAs assays sensitivity for the diagnosis of CNS cancers. miRNAs in CSF showed higher levels in sensitivity of CNS cancers detection suggesting a relatively high diagnostic accuracy specially for PCNSL. By the end of the study, the authors concluded that CSF based miRNAs assays may be suitable as biomarkers for detection of CNS cancers and could be considered more reliable for clinical application [108].

At the time of writing this manuscript, Drusco *et al.* [61] analyzed 82 CFS samples from neoplastic and non-diseased patients (normal, benign, glioblastoma, medulloblastoma, metastasis and lymphoma) by Nanostring technique to identify a CSF microRNA signature that could differentiate among CNS malignancies. The authors found that miR-451, -711, 935, -223 and -125b are significantly differentially expressed in CSF of the tested groups and can differentiate between some classes of CNS tumors tested. Their miRNA profile was further confirmed by RT-PCR and *in situ* hybridization. Based on their results, the authors proposed a hypothetical diagnostic chart for CNS malignancies with a simple RT-PCR on patients’ CSF miRNAs. However, such miRNA signatures needs to be tested on more samples with additional pathology and prognostic classes to determine differentiating ranges of fold changes of expression among groups.

## 6. Considerations and Concerns about the Use of Circulating miRNAs as Biomarkers

An accurate non-invasive diagnostic test for brain tumors is unavailable; moreover, the current diagnostic tools have limitations and under-diagnosis remains a major problem. Therefore, the goal is to search for biomarkers that are able to detect early indicative sign of CNS neoplastic presence in order to enable clinicians to react early with the most relevant therapy [109]. Early histological diagnosis could be made possible by analysis for biomarkers specific to particular types of brain tumor [110]. This would aid prognostication and help direct pre-operative management, such as chemotherapy and radiotherapy. miRNAs circulating in the CSF of brain cancer patients offer a dynamic and powerful approach to understanding the spectrum of brain cancers from the earliest manifestations to the terminal stages. Their analysis provides insights about disease biology genetic changes that might help to decode the molecular features of the underlying malignancy [87]. CSF miRNAs specificity and chemical stability are clinically appealing as they are easily accessible by minimally-invasive standard clinical methods [90,111]. Only small amounts of CSF samples are usually required for the detection of miRNAs in the CSF, depending on which detection methods are used. This offers the advantage of convenient repetitive monitoring of molecular events happening in cancer in the response to treatment.

The rapid growth of miRNAs’ identification techniques and measuring technology, such as qRT-PCR, next-generation sequencing or microarrays, gives us hope that the application of miRNAs in CSF as technically feasible biomarkers will soon become clinically practicable. There are great expectations that detecting miRNAs in CSF of brain tumor patients will achieve enhanced clinical utility in the near future and will lead to the identification of individuals in the “preclinical” stages of the illness. However, there are several concerns to consider before recommending the clinical use of CSF miRNAs as markers for brain tumors.

**Figure 3:**
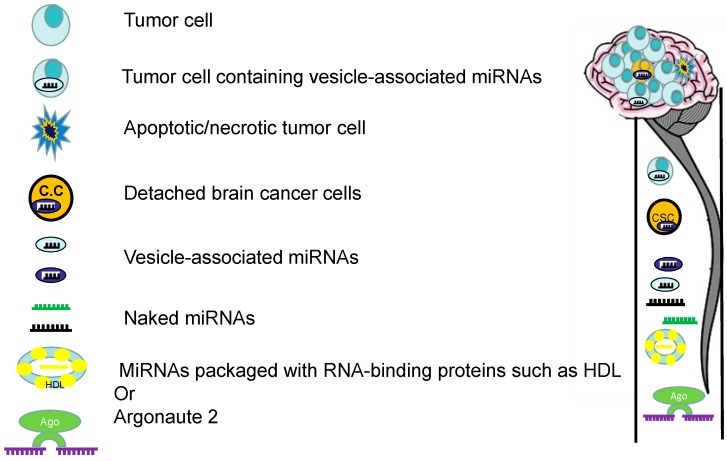
Depiction of hypothetical origins and types of miRNAs in the CSF: Brain tumor-associated miRNAs in the CSF could be actively released by brain tumor cells to the CSF either enclosed in small membranous microvesicles (e.g., exosomes) or packaged with RNA-binding proteins, e.g., high-density lipoprotein (HDL) or Argonaute (Ago). A second possibility is that miRNAs are passively released in the CSF by dead/dying cancer cells where it remains stably complexed to Ago in the extracellular environment. In addition, freely circulating naked miRNAs (free of exosomes or microvesicles) might be actively secreted from brain tumor cells or passively released from apoptotic or necrotic cells. A third possibility is that a proportion of miRNAs are likely associated with detached brain cancer cells (C.C) that are circulating in the CSF.

The first concern is that little is known about the origin of c-miRNAs in the CSF of brain tumor patients, what factors influence their level of expression and what impact this will have on their specificity as biomarkers. The stability of circulating miRNAs in body fluids and culture media of some cell lines suggests that they are likely packaged in some manner that protects them against RNase digestion [89]. Studies have suggested that the majority of these miRNAs are enclosed in lipid vesicles such as xosomes and microvesicles or in complexes with RNA-binding proteins lines [18,112,113] (Figure 3). It has been hypothesized recently that such actively secreted exosomal miRNAs are involved in intercellular communication [16,17,18]. However, it remains unclear whether c-miRNAs are present in physiologically relevant amounts for cell-to-cell signaling. In contrast, other studies suggest that most of the miRNAs in body fluids might be an offshoot of dead/dying cells and they showed that such c-miRNAs are microvesicles free and independent from exosomes [19,114]. In this scenario, c-miRNAs are bound to Argonaute (Ago) proteins as part of RNA-induced silencing complex, which remain in extracellular space due to the high stability of the miRNA/Ago complex [21]. However, the secretory mechanism and biological function, as well as the meaning of the existence of extracellular miRNAs, remains largely unclear.

The ideal scenario is that CSF c-miRNAs are cancer related, originated from brain cancer cells in the primary tumor and accordingly reflect the magnitude of the disease. This will lead to the second concern that the chance is high that c-miRNAs in the CSF of brain tumor patients could be expressed from or secreted by a mixture of different cell types in the tumor microenvironment, such as inflammatory cells or other physiological response against the diseased tissue, jeopardizing their specificity and their ability to honestly represent tumor cells [115]. The third concern is that levels of miRNAs do not only depend on the production rate of cancer cell populations but can also be influenced by many other factors, including, cell degradation rates, clearance by liver and kidney, infection, age, sex, treatment, epigenetic mechanisms, diet, lifestyle, and more [116].

Estimates of miRNA expression levels are known to be significantly affected by methods of analysis and different miRNA profiles may be obtained depending on the sample analysis used. However, there are no universally implemented guidelines for CSF sample preparation, miRNAs extraction, measurements, normalization, or data analysis. On the contrary, there are various platforms/techniques which exist, each with specific biases that can influence the results of certain miRNA molecules expression measurements in the tested sample and may lead to foregone.

It is not surprising that there are often low correlations of results between different labs and, hence, standardization is a challenge for the near future. The fourth concern is related to technical challenges associated with miRNAs analysis.

### 6.1. Issues Associated with CSF Collection and Preparation

Collection of CSF is the first step in sample preparation; however, CSF is not an easy specimen to obtain compared to other body fluids. Lumbar puncture (LP), although it poses a minimal risk to the patient compared to biopsy, major and minor complications can occur even when standard infection control measures and good techniques are used. These complications include: post-LP headache, infection, bleeding, cerebral herniation and back pain.

Another worry is that CSF samples that are used as material for miRNA detection could be contaminated with blood iatrogenically. Blood contamination of CSF would result in significant increases in nucleated white blood cells (WBC) count that could contribute to miRNA content of the sample being analyzed. Skin cells can also contaminate CSF samples. In this situation, skin contains abundant epithelial miRNAs that can bias results of miRNAs profiles. Method that preserves the RNA expression profile during and after collection of CSF is another important factor for accurate analysis of miRNA expression [117]. Centrifugation to remove debris or precipitates from CSF samples prior to RNA extraction will alter miRNA profiles if it is not performed accurately. Currently, there are no standardized centrifugation conditions (time, temperature, g-force, rotor type) to be used to prepare cell-free miRNAs from CSF samples as residual cell debris can alter miRNA abundance [118]. Sample freezing condition (whether snap or slow freezing) and storage time and temperature should also be standardized.

### 6.2. Issues Associated with miRNA Isolation and Assessment

In general, working with RNA requires very special precautions to prevent degradation and or contamination of the RNA sample. The practice for isolating miRNAs are, in principle, the same as for isolation of total RNA, except that miRNA isolation protocols are often slightly modified to retain the miRNAs. The first challenge is that CSF samples contain only small amounts of RNA and extraction of sufficient miRNA amounts can be difficult [27]. Samples with low miRNA abundance create challenges for downstream assays. Appropriate assessment of the quality and quantity of extracted RNA is important for reproducibility and accuracy in miRNA-profiling studies. That will bring us to the second challenge where quantification and measuring the amount of miRNAs isolated from CSF samples is especially difficult due to the low concentration of RNA typically obtained from these samples [117]. The low abundance of miRNA in CSF can hardly be determined using standard simple methods for measurement of the RNA quantity and quality such as Nano-drop or other spectrophotometers. Hence, an alternative multifaceted way to assess the yield and RNA integrity has to be used. In this case, automated capillary electrophoresis instruments could be used [26,37]. Finally, in addition to low miRNA concentration, samples are likely to contain high levels of enzyme inhibitors that might affect the efficiency of the reverse transcription and choosing a purification method that minimizes the effect of such inhibitors is still a great challenge.

### 6.3. Issues Associated with miRNA-Profiling/Detection Methods

Three major approaches are currently used for miRNAs detection in the CSF: qRT-PCR, hybridization-based methods, such as DNA microarrays, and high-throughput sequencing (RNA-seq). qRT-PCR assays are among the most frequently used high-throughput RT-PCR platforms for miRNA detection. The attractive aspect of this approach is the ease of incorporation into the workflow for laboratories that are familiar with real-time PCR. However, commercial assays that are commonly used for miRNA detection use a different strategy to reverse transcribe mature miRNA molecules and to amplify the cDNA, which might result in different detection biases. Another obstacle in performing qRT-PCR is that reaction conditions may vary as well as sequence-specific differences in primers [117].

### 6.4. Issues Associated with Data Normalization

Finally, data normalization (*i.e.*, adjusting the data and removing technical variations across samples that are not related to the biological changes under investigation) is tricky and can be challenging. The optimal method to use for normalization is to identify stably expressed reference genes or commonly expressed miRNAs in a given sample as normalization factor. However, no constitutively expressed circulating miRNAs have been reported up till now in the CSF. Spiking samples with RNA controls, applying reference miRNA such as miR-16 or miR-26a or other non-coding RNA like U6 snRNA are some of the strategies used to standardize analysis, however some of the larger small RNA species such as U6 RNA are present in extremely low concentrations in body fluids. On the whole, the variability in normalization methods and analysis might be responsible for some of the discrepancies between miRNA-profiling studies and differences and overlapped miRNAs signatures reported in the literature.

In summary, miRNA investigation in CSF samples as a potential marker for brain cancers is a challenging procedure. The development of miRNAs as good biomarkers requires optimized and standardized procedures for CSF sample collection handling and preparation, highly sensitive and accurate miRNAs detection methods as well as reliable methods for data analysis [115].

## 7. Conclusions

Tumor-associated miRNAs circulating in the CSF have recently shown promise as diagnostic indicators for some types of brain tumors owing to the substantial differences that have been described between their expression profiles in healthy individuals and patients. Their chemical stability in the CSF together with the recent advances in the sensitive mRNA measuring platforms have generated demand to apply CSF miRNAs investigation procedure in clinical practice. However, despite the promise, the available data on the suitability of the CSF miRNAs as diagnostic biomarkers for brain cancer are still very limited and critical issues need to be clarified before making recommendations for their clinical implementation. We need to have a better understanding about the mechanisms by which tumor-specific miRNAs are produced in the CSF, what factors influence their level of expression in the CSF of patients with brain tumors, and what impact will this have on their specificity as diagnostic markers. More studies are certainly necessary to establish miRNAs signatures for each brain cancer type and their different stages, and to determine which miRNAs meaningfully reflect the dynamics of tumor response to therapy. Undoubtedly, CSF miRNAs are a potential gold mine for identifying brain cancer biomarkers, however, much work remains to be done to harness findings from scientific research to advance clinical applications.

## References

[1] Nayak L., Lee E.Q., Wen P.Y. (2012). Epidemiology of brain metastases. Curr. Oncol. Rep..

[2] Teplyuk N.M., Mollenhauer B., Gabriely G., Giese A., Kim E., Smolsky M., Kim R.Y., Saria M.G., Pastorino S., Kesari S. (2012). MicroRNAs in cerebrospinal fluid identify glioblastoma and metastatic brain cancers and reflect disease activity. Neuro-Oncology.

[3] Astrakas L.G., Zurakowski D., Tzika A.A., Zarifi M.K., Anthony D.C., de Girolami U., Tarbell N.J., Black P.M. (2004). Noninvasive magnetic resonance spectroscopic imaging biomarkers to predict the clinical grade of pediatric brain tumors. Clin. Cancer Res..

[4] Gomes H.R. (2013). Cerebrospinal fluid approach on neuro-oncology. Arq. Neuropsiquiatr..

[5] Yotsukura S., Mamitsuka H. (2015). Evaluation of serum-based cancer biomarkers: A brief review from a clinical and computational viewpoint. Crit. Rev. Oncol. Hematol..

[6] Alsidawi S., Malek E., Driscoll J.J. (2014). MicroRNAs in brain metastases: Potential role as diagnostics and therapeutics. Int. J. Mol. Sci..

[7] Mishra P.J. (2012). The miRNA-drug resistance connection: A new era of personalized medicine using noncoding RNA begins. Pharmacogenomics.

[8] Cortez M.A., Bueso-Ramos C., Ferdin J., Lopez-Berestein G., Sood A.K., Calin G.A. (2011). MicroRNAs in body fluids—The mix of hormones and biomarkers. Nat. Rev. Clin. Oncol..

[9] Allegra A., Alonci A., Campo S., Penna G., Petrungaro A., Gerace D., Musolino C. (2012). Circulating microRNAs: New biomarkers in diagnosis, prognosis and treatment of cancer (review). Int. J. Oncol..

[10] D'Asti E., Garnier D., Lee T.H., Montermini L., Meehan B., Rak J. (2012). Oncogenic extracellular vesicles in brain tumor progression. Front. Physiol..

[11] Chen X., Ba Y., Ma L., Cai X., Yin Y., Wang K., Guo J., Zhang Y., Chen J., Guo X. (2008). Characterization of microRNAs in serum: A novel class of biomarkers for diagnosis of cancer and other diseases. Cell Res..

[12] Cogswell J.P., Ward J., Taylor I.A., Waters M., Shi Y., Cannon B., Kelnar K., Kemppainen J., Brown D., Chen C. (2008). Identification of miRNA changes in Alzheimer’s disease brain and CSF yields putative biomarkers and insights into disease pathways. J. Alzheimer's Dis..

[13] Melkonyan H.S., Feaver W.J., Meyer E., Scheinker V., Shekhtman E.M., Xin Z., Umansky S.R. (2008). Transrenal nucleic acids: From proof of principle to clinical tests. Ann. N. Y. Acad. Sci..

[14] Mitchell P.S., Parkin R.K., Kroh E.M., Fritz B.R., Wyman S.K., Pogosova-Agadjanyan E.L., Peterson A., Noteboom J., O’Briant K.C., Allen A. (2008). Circulating microRNAs as stable blood-based markers for cancer detection. Proc. Natl. Acad. Sci. USA.

[15] Park N.J., Zhou H., Elashoff D., Henson B.S., Kastratovic D.A., Abemayor E., Wong D.T. (2009). Salivary microRNA: Discovery, characterization, and clinical utility for oral cancer detection. Clin. Cancer Res..

[16] Kosaka N., Iguchi H., Ochiya T. (2010). Circulating microRNA in body fluid: A new potential biomarker for cancer diagnosis and prognosis. Cancer Sci..

[17] Kosaka N., Iguchi H., Yoshioka Y., Takeshita F., Matsuki Y., Ochiya T. (2010). Secretory mechanisms and intercellular transfer of microRNAs in living cells. J. Biol. Chem..

[18] Valadi H., Ekstrom K., Bossios A., Sjostrand M., Lee J.J., Lotvall J.O. (2007). Exosome-mediated transfer of mRNAs and microRNAs is a novel mechanism of genetic exchange between cells. Nat. Cell Biol..

[19] Wang K., Zhang S., Weber J., Baxter D., Galas D.J. (2010). Export of microRNAs and microRNA-protective protein by mammalian cells. Nucleic Acids Res..

[20] Lukiw W.J., Alexandrov P.N., Zhao Y., Hill J.M., Bhattacharjee S. (2010). Spreading of Alzheimer’s disease inflammatory signaling through soluble micro-RNA. NeuroReport.

[21] Turchinovich A., Weiz L., Langheinz A., Burwinkel B. (2011). Characterization of extracellular circulating microRNA. Nucleic Acids Res..

[22] Shalaby T.F.G., Baulande S., Gerber N.U., Baumgartner M., Grotzer M.A. (2015). Detection and quantification of extracellular microRNAs in medulloblastoma. J. Cancer Metastasis Treat..

[23] Denk J., Boelmans K., Siegismund C., Lassner D., Arlt S., Jahn H. (2015). MicroRNA profiling of CSF reveals potential biomarkers to detect Alzheimer’s disease. PLoS ONE.

[24] Jin X.F., Wu N., Wang L., Li J. (2013). Circulating microRNAs: A novel class of potential biomarkers for diagnosing and prognosing central nervous system diseases. Cell. Mol. Neurobiol..

[25] Baraniskin A., Kuhnhenn J., Schlegel U., Maghnouj A., Zollner H., Schmiegel W., Hahn S., Schroers R. (2012). Identification of microRNAs in the cerebrospinal fluid as biomarker for the diagnosis of glioma. Neuro-Oncology.

[26] Shalaby T., Fiaschetti G., Baumgartner M., Grotzer M.A. (2014). Significance and therapeutic value of miRNAs in embryonal neural tumors. Molecules.

[27] Burgos K.L., Javaherian A., Bomprezzi R., Ghaffari L., Rhodes S., Courtright A., Tembe W., Kim S., Metpally R., van Keuren-Jensen K. (2013). Identification of extracellular miRNA in human cerebrospinal fluid by next-generation sequencing. RNA.

[28] Hesse M., Arenz C. (2014). miRNAs as novel therapeutic targets and diagnostic biomarkers for Parkinson’s disease: A patent evaluation of WO2014018650. Expert Opin. Ther. Pat..

[29] Scott B.J., Douglas V.C., Tihan T., Rubenstein J.L., Josephson S.A. (2013). A systematic approach to the diagnosis of suspected central nervous system lymphoma. JAMA Neurol..

[30] Sorensen S.S., Nygaard A.B., Nielsen M.Y., Jensen K., Christensen T. (2014). miRNA expression profiles in cerebrospinal fluid and blood of patients with acute ischemic stroke. Transl. Stroke Res..

[31] Lawrie C.H., Gal S., Dunlop H.M., Pushkaran B., Liggins A.P., Pulford K., Banham A.H., Pezzella F., Boultwood J., Wainscoat J.S. (2008). Detection of elevated levels of tumour-associated microRNAs in serum of patients with diffuse large B-cell lymphoma. Br. J. Haematol..

[32] Hu Z., Chen X., Zhao Y., Tian T., Jin G., Shu Y., Chen Y., Xu L., Zen K., Zhang C. (2010). Serum microRNA signatures identified in a genome-wide serum microRNA expression profiling predict survival of non-small-cell lung cancer. J. Clin. Oncol..

[33] Ng E.K., Chong W.W., Jin H., Lam E.K., Shin V.Y., Yu J., Poon T.C., Ng S.S., Sung J.J. (2009). Differential expression of microRNAs in plasma of patients with colorectal cancer: A potential marker for colorectal cancer screening. Gut.

[34] Yamamoto Y., Kosaka N., Tanaka M., Koizumi F., Kanai Y., Mizutani T., Murakami Y., Kuroda M., Miyajima A., Kato T. (2009). MicroRNA-500 as a potential diagnostic marker for hepatocellular carcinoma. Biomarkers.

[35] Wong T.S., Liu X.B., Wong B.Y., Ng R.W., Yuen A.P., Wei W.I. (2008). Mature miR-184 as potential oncogenic microRNA of squamous cell carcinoma of tongue. Clin. Cancer Res..

[36] Grunder E., D’Ambrosio R., Fiaschetti G., Abela L., Arcaro A., Zuzak T., Ohgaki H., Lv S.Q., Shalaby T., Grotzer M. (2011). MicroRNA-21 suppression impedes medulloblastoma cell migration. Eur. J. Cancer.

[37] Shalaby T., Fiaschetti G., Baumgartner M., Grotzer M.A. (2014). MicroRNA signatures as biomarkers and therapeutic target for CNS embryonal tumors: the pros and the cons. Int. J. Mol. Sci..

[38] Fiaschetti G., Abela L., Nonoguchi N., Dubuc A.M., Remke M., Boro A., Grunder E., Siler U., Ohgaki H., Taylor M.D. (2014). Epigenetic silencing of miRNA-9 is associated with HES1 oncogenic activity and poor prognosis of medulloblastoma. Br. J. Cancer.

[39] Hennessey P.T., Sanford T., Choudhary A., Mydlarz W.W., Brown D., Adai A.T., Ochs M.F., Ahrendt S.A., Mambo E., Califano J.A. (2012). Serum microRNA biomarkers for detection of non-small cell lung cancer. PLoS ONE.

[40] Cuk K., Zucknick M., Heil J., Madhavan D., Schott S., Turchinovich A., Arlt D., Rath M., Sohn C., Benner A. (2013). Circulating microRNAs in plasma as early detection markers for breast cancer. Int. J. Cancer.

[41] Xu Y.Z., Xi Q.H., Ge W.L., Zhang X.Q. (2013). Identification of serum microRNA-21 as a biomarker for early detection and prognosis in human epithelial ovarian cancer. Asian Pac. J. Cancer Prev..

[42] Yu J., Wang Y., Dong R., Huang X., Ding S., Qiu H. (2012). Circulating microRNA-218 was reduced in cervical cancer and correlated with tumor invasion. J. Cancer Res. Clin. Oncol..

[43] Cheng H.H., Mitchell P.S., Kroh E.M., Dowell A.E., Chery L., Siddiqui J., Nelson P.S., Vessella R.L., Knudsen B.S., Chinnaiyan A.M. (2013). Circulating microRNA profiling identifies a subset of metastatic prostate cancer patients with evidence of cancer-associated hypoxia. PLoS ONE.

[44] Redova M., Poprach A., Nekvindova J., Iliev R., Radova L., Lakomy R., Svoboda M., Vyzula R., Slaby O. (2012). Circulating miR-378 and miR-451 in serum are potential biomarkers for renal cell carcinoma. J. Transl. Med..

[45] Kanaan Z., Roberts H., Eichenberger M.R., Billeter A., Ocheretner G., Pan J., Rai S.N., Jorden J., Williford A., Galandiuk S. (2013). A plasma microRNA panel for detection of colorectal adenomas: A step toward more precise screening for colorectal cancer. Ann. Surg..

[46] Liu H., Zhu L., Liu B., Yang L., Meng X., Zhang W., Ma Y., Xiao H. (2012). Genome-wide microRNA profiles identify miR-378 as a serum biomarker for early detection of gastric cancer. Cancer Lett..

[47] Tan Y., Ge G., Pan T., Wen D., Chen L., Yu X., Zhou X., Gan J. (2014). A serum microRNA panel as potential biomarkers for hepatocellular carcinoma related with hepatitis B virus. PLoS ONE.

[48] Liu R., Chen X., Du Y., Yao W., Shen L., Wang C., Hu Z., Zhuang R., Ning G., Zhang C. (2012). Serum microRNA expression profile as a biomarker in the diagnosis and prognosis of pancreatic cancer. Clin. Chem..

[49] Kurashige J., Kamohara H., Watanabe M., Tanaka Y., Kinoshita K., Saito S., Hiyoshi Y., Iwatsuki M., Baba Y., Baba H. (2012). Serum microRNA-21 is a novel biomarker in patients with esophageal squamous cell carcinoma. J. Surg. Oncol..

[50] Hsu C.M., Lin P.M., Wang Y.M., Chen Z.J., Lin S.F., Yang M.Y. (2012). Circulating miRNA is a novel marker for head and neck squamous cell carcinoma. Tumour Biol..

[51] Cantara S., Pilli T., Sebastiani G., Cevenini G., Busonero G., Cardinale S., Dotta F., Pacini F. (2014). Circulating miRNA95 and miRNA190 are sensitive markers for the differential diagnosis of thyroid nodules in a Caucasian population. J. Clin. Endocrinol. Metab..

[52] Kanemaru H., Fukushima S., Yamashita J., Honda N., Oyama R., Kakimoto A., Masuguchi S., Ishihara T., Inoue Y., Jinnin M. (2011). The circulating microRNA-221 level in patients with malignant melanoma as a new tumor marker. J. Dermatol. Sci..

[53] Ayaz L., Gorur A., Yaroglu H.Y., Ozcan C., Tamer L. (2013). Differential expression of microRNAs in plasma of patients with laryngeal squamous cell carcinoma: Potential early-detection markers for laryngeal squamous cell carcinoma. J. Cancer Res. Clin. Oncol..

[54] Guo H.Q., Huang G.L., Guo C.C., Pu X.X., Lin T.Y. (2010). Diagnostic and prognostic value of circulating miR-221 for extranodal natural killer/T-cell lymphoma. Dis. Markers.

[55] Ferrajoli A., Shanafelt T.D., Ivan C., Shimizu M., Rabe K.G., Nouraee N., Ikuo M., Ghosh A.K., Lerner S., Rassenti L.Z. (2013). Prognostic value of miR-155 in individuals with monoclonal B-cell lymphocytosis and patients with B chronic lymphocytic leukemia. Blood.

[56] Wang Q., Li P., Li A., Jiang W., Wang H., Wang J., Xie K. (2012). Plasma specific miRNAs as predictive biomarkers for diagnosis and prognosis of glioma. J. Exp. Clin. Cancer Res..

[57] Ilhan-Mutlu A., Wagner L., Wohrer A., Furtner J., Widhalm G., Marosi C., Preusser M. (2012). Plasma MicroRNA-21 concentration may be a useful biomarker in glioblastoma patients. Cancer Investig..

[58] Ilhan-Mutlu A., Wagner L., Wohrer A., Jungwirth S., Marosi C., Fischer P., Preusser M. (2012). Blood alterations preceding clinical manifestation of glioblastoma. Cancer Investig..

[59] Yang C., Wang C., Chen X., Chen S., Zhang Y., Zhi F., Wang J., Li L., Zhou X., Li N. (2013). Identification of seven serum microRNAs from a genome-wide serum microRNA expression profile as potential noninvasive biomarkers for malignant astrocytomas. Int. J. Cancer.

[60] Shi R., Wang P.Y., Li X.Y., Chen J.X., Li Y., Zhang X.Z., Zhang C.G., Jiang T., Li W.B., Ding W. (2015). Exosomal levels of miRNA-21 from cerebrospinal fluids associated with poor prognosis and tumor recurrence of glioma patients. Oncotarget.

[61] Drusco A., Bottoni A., Lagana A., Acunzo M., Fassan M., Cascione L., Antenucci A., Kumchala P., Vicentini C., Gardiman M.P. (2015). A differentially expressed set of microRNAs in cerebro-spinal fluid (CSF) can diagnose CNS malignancies. Oncotarget.

[62] Baraniskin A., Kuhnhenn J., Schlegel U., Chan A., Deckert M., Gold R., Maghnouj A., Zollner H., Reinacher-Schick A., Schmiegel W. (2011). Identification of microRNAs in the cerebrospinal fluid as marker for primary diffuse large B-cell lymphoma of the central nervous system. Blood.

[63] Machida A., Ohkubo T., Yokota T. (2013). Circulating microRNAs in the cerebrospinal fluid of patients with brain diseases. Methods Mol. Biol..

[64] Samuel N., Remke M., Rutka J.T., Raught B., Malkin D. (2014). Proteomic analyses of CSF aimed at biomarker development for pediatric brain tumors. J. Neurooncol..

[65] Weston C.L., Glantz M.J., Connor J.R. (2011). Detection of cancer cells in the cerebrospinal fluid: Current methods and future directions. Fluids Barriers CNS.

[66] Patel A.S., Allen J.E., Dicker D.T., Peters K.L., Sheehan J.M., Glantz M.J., El-Deiry W.S. (2011). Identification and enumeration of circulating tumor cells in the cerebrospinal fluid of breast cancer patients with central nervous system metastases. Oncotarget.

[67] Baraniskin A., Schroers R. (2014). Modern cerebrospinal fluid analyses for the diagnosis of diffuse large B-cell lymphoma of the CNS. CNS Oncol..

[68] Roy S., Josephson S.A., Fridlyand J., Karch J., Kadoch C., Karrim J., Damon L., Treseler P., Kunwar S., Shuman M.A. (2008). Protein biomarker identification in the CSF of patients with CNS lymphoma. J. Clin. Oncol..

[69] Schroers R., Baraniskin A., Heute C., Kuhnhenn J., Alekseyev A., Schmiegel W., Schlegel U., Pels H.J. (2010). Detection of free immunoglobulin light chains in cerebrospinal fluids of patients with central nervous system lymphomas. Eur. J. Haematol..

[70] Bougel S., Lhermitte B., Gallagher G., de Flaugergues J.C., Janzer R.C., Benhattar J. (2013). Methylation of the hTERT promoter: A novel cancer biomarker for leptomeningeal metastasis detection in cerebrospinal fluids. Clin. Cancer Res..

[71] Galati D., di Noto R., del Vecchio L. (2013). Diagnostic strategies to investigate cerebrospinal fluid involvement in haematological malignancies. Leuk. Res..

[72] Huttner A. (2012). Overview of primary brain tumors: Pathologic classification, epidemiology, molecular biology, and prognostic markers. Hematol. Oncol. Clin. N. Am..

[73] Kersten M.J., Evers L.M., Dellemijn P.L., van den Berg H., Portegies P., Hintzen R.Q., van Lier R.A., von dem Borne A.E., van Oers R.H. (1996). Elevation of cerebrospinal fluid soluble CD27 levels in patients with meningeal localization of lymphoid malignancies. Blood.

[74] Rubenstein J.L., Wong V.S., Kadoch C., Gao H.X., Barajas R., Chen L., Josephson S.A., Scott B., Douglas V., Maiti M. (2013). CXCL13 plus interleukin 10 is highly specific for the diagnosis of CNS lymphoma. Blood.

[75] Chamberlain M.C., Johnston S.K. (2009). Neoplastic meningitis: Survival as a function of cerebrospinal fluid cytology. Cancer.

[76] Kros J.M., Mustafa D.M., Dekker L.J., Sillevis Smitt P.A., Luider T.M., Zheng P.P. (2015). Circulating glioma biomarkers. Neuro-Oncology.

[77] Liu B.L., Cheng J.X., Zhang W., Zhang X., Wang R., Lin H., Huo J.L., Cheng H. (2010). Quantitative detection of multiple gene promoter hypermethylation in tumor tissue, serum, and cerebrospinal fluid predicts prognosis of malignant gliomas. Neuro-Oncology.

[78] Chamberlain M.C. (2010). Leptomeningeal metastasis. Curr. Opin. Oncol..

[79] Gleissner B., Chamberlain M.C. (2006). Neoplastic meningitis. Lancet Neurol..

[80] Le Rhun E., Taillibert S., Chamberlain M.C. (2013). Carcinomatous meningitis: Leptomeningeal metastases in solid tumors. Surg. Neurol. Int..

[81] Lai N.S., Wu D.G., Fang X.G., Lin Y.C., Chen S.S., Li Z.B., Xu S.S. (2015). Serum microRNA-210 as a potential noninvasive biomarker for the diagnosis and prognosis of glioma. Br. J. Cancer.

[82] Zetterberg H., Andreasson U., Blennow K. (2009). CSF antithrombin III and disruption of the blood-brain barrier. J. Clin. Oncol..

[83] Yang H., Cai L., Zhang Y., Tan H., Deng Q., Zhao M., Xu X. (2014). Sensitive detection of EGFR mutations in cerebrospinal fluid from lung adenocarcinoma patients with brain metastases. J. Mol. Diagn..

[84] Albulescu R., Codrici E., Popescu I.D., Mihai S., Necula L.G., Petrescu D., Teodoru M., Tanase C.P. (2013). Cytokine patterns in brain tumour progression. Mediat. Inflamm..

[85] Wang P., Piao Y., Zhang X., Li W., Hao X. (2013). The concentration of CYFRA 21-1, NSE and CEA in cerebro-spinal fluid can be useful indicators for diagnosis of meningeal carcinomatosis of lung cancer. Cancer Biomark..

[86] Tang Y.T., Jiang F., Guo L., Si M.Y., Jiao X.Y. (2013). The soluble VEGF receptor 1 and 2 expression in cerebral spinal fluid as an indicator for leukemia central nervous system metastasis. J. Neurooncol..

[87] Yue X., Lan F., Hu M., Pan Q., Wang Q., Wang J. (2015). Downregulation of serum microRNA-205 as a potential diagnostic and prognostic biomarker for human glioma. J. Neurosurg..

[88] Kiviniemi A., Gardberg M., Frantzen J., Parkkola R., Vuorinen V., Pesola M., Minn H. (2015). Serum levels of GFAP and EGFR in primary and recurrent high-grade gliomas: Correlation to tumor volume, molecular markers, and progression-free survival. J. Neurooncol..

[89] Sasayama T., Nakamizo S., Nishihara M., Kawamura A., Tanaka H., Mizukawa K., Miyake S., Taniguchi M., Hosoda K., Kohmura E. (2012). Cerebrospinal fluid interleukin-10 is a potentially useful biomarker in immunocompetent primary central nervous system lymphoma (PCNSL). Neuro-Oncology.

[90] Rajagopal M.U., Hathout Y., MacDonald T.J., Kieran M.W., Gururangan S., Blaney S.M., Phillips P., Packer R., Gordish-Dressman H., Rood B.R. (2011). Proteomic profiling of cerebrospinal fluid identifies prostaglandin D2 synthase as a putative biomarker for pediatric medulloblastoma: A pediatric brain tumor consortium study. Proteomics.

[91] Pantazis G., Psaras T., Krope K., von Coelln R., Fend F., Bock T., Schittenhelm J., Melms A., Meyermann R., Bornemann A. (2010). Cerebral low-grade lymphoma and light chain deposition disease: Exceedingly high IgG levels in the cerebrospinal fluid as a diagnostic clue. Clin. Neuropathol..

[92] Fischer L., Korfel A., Pfeiffer S., Kiewe P., Volk H.D., Cakiroglu H., Widmann T., Thiel E. (2009). CXCL13 and CXCL12 in central nervous system lymphoma patients. Clin. Cancer Res..

[93] Shnaper S., Desbaillets I., Brown D.A., Murat A., Migliavacca E., Schluep M., Ostermann S., Hamou M.F., Stupp R., Breit S.N. (2009). Elevated levels of MIC-1/GDF15 in the cerebrospinal fluid of patients are associated with glioblastoma and worse outcome. Int. J. Cancer.

[94] Groves M.D., Hess K.R., Puduvalli V.K., Colman H., Conrad C.A., Gilbert M.R., Weinberg J., Cristofanilli M., Yung W.K., Liu T.J. (2009). Biomarkers of disease: Cerebrospinal fluid vascular endothelial growth factor (VEGF) and stromal cell derived factor (SDF)-1 levels in patients with neoplastic meningitis (NM) due to breast cancer, lung cancer and melanoma. J. Neurooncol..

[95] De Bont J.M., den Boer M.L., Reddingius R.E., Jansen J., Passier M., van Schaik R.H., Kros J.M., Sillevis Smitt P.A., Luider T.H., Pieters R. (2006). Identification of apolipoprotein A-II in cerebrospinal fluid of pediatric brain tumor patients by protein expression profiling. Clin. Chem..

[96] Herrlinger U., Wiendl H., Renninger M., Forschler H., Dichgans J., Weller M. (2004). Vascular endothelial growth factor (VEGF) in leptomeningeal metastasis: Diagnostic and prognostic value. Br. J. Cancer.

[97] Sampath P., Weaver C.E., Sungarian A., Cortez S., Alderson L., Stopa E.G. (2004). Cerebrospinal fluid (vascular endothelial growth factor) and serologic (recoverin) tumor markers for malignant glioma. Cancer Control.

[98] Wong L.J., Lueth M., Li X.N., Lau C.C., Vogel H. (2003). Detection of mitochondrial DNA mutations in the tumor and cerebrospinal fluid of medulloblastoma patients. Cancer Res..

[99] Miyanohara O., Takeshima H., Kaji M., Hirano H., Sawamura Y., Kochi M., Kuratsu J. (2002). Diagnostic significance of soluble c-kit in the cerebrospinal fluid of patients with germ cell tumors. J. Neurosurg..

[100] Seregni E., Massimino M., Nerini Molteni S., Pallotti F., van der Hiel B., Cefalo G., Spreafico F., Fossati F., Bombardieri E. (2002). Serum and cerebrospinal fluid human chorionic gonadotropin (hCG) and α-fetoprotein (AFP) in intracranial germ cell tumors. Int. J. Biol. Markers.

[101] Kawashima M., Suzuki S.O., Yamashima T., Fukui M., Iwaki T. (2001). Prostaglandin D synthase (β-trace) in meningeal hemangiopericytoma. Mod. Pathol..

[102] Saso L., Leone M.G., Sorrentino C., Giacomelli S., Silvestrini B., Grima J., Li J.C., Samy E., Mruk D., Cheng C.Y. (1998). Quantification of prostaglandin D synthetase in cerebrospinal fluid: A potential marker for brain tumor. Biochem. Mol. Biol. Int..

[103] Murase S., Saio M., Andoh H., Takenaka K., Shinoda J., Nishimura Y., Sakai N., Takami T. (2000). Diagnostic utility of CSF soluble CD27 for primary central nervous system lymphoma in immunocompetent patients. Neurol. Res..

[104] Bakri Y., al-Hawashim N., Berkowitz R. (2000). CSF/serum β-hCG ratio in patients with brain metastases of gestational trophoblastic tumor. J. Reprod. Med..

[105] Hannafon B.N., Ding W.Q. (2013). Intercellular communication by exosome-derived microRNAs in cancer. Int. J. Mol. Sci..

[106] Mittelbrunn M., Sanchez-Madrid F. (2012). Intercellular communication: Diverse structures for exchange of genetic information. Nat. Rev. Mol. Cell Biol..

[107] Snuderl M., Batista A., Kirkpatrick N.D., Ruiz de Almodovar C., Riedemann L., Walsh E.C., Anolik R., Huang Y., Martin J.D., Kamoun W. (2013). Targeting placental growth factor/neuropilin 1 pathway inhibits growth and spread of medulloblastoma. Cell.

[108] Wei D., Wan Q., Li L., Jin H., Liu Y., Wang Y., Zhang G. (2015). MicroRNAs as potential biomarkers for diagnosing cancers of central nervous system: A meta-analysis. Mol. Neurobiol..

[109] Ajit S.K. (2010). Circulating microRNAs as biomarkers, therapeutic targets, and signaling molecules. Sensors.

[110] Russell M.D., Young A.M., Karri S.K. (2013). Biomarkers of pediatric brain tumors. Front. Pediatr..

[111] Leach P.A., Estlin E.J., Coope D.J., Thorne J.A., Kamaly-Asl I.D. (2008). Diffuse brainstem gliomas in children: Should we or shouldn’t we biopsy?. Br. J. Neurosurg..

[112] Hunter M.P., Ismail N., Zhang X., Aguda B.D., Lee E.J., Yu L., Xiao T., Schafer J., Lee M.L., Schmittgen T.D. (2008). Detection of microRNA expression in human peripheral blood microvesicles. PLoS ONE.

[113] Mathivanan S., Ji H., Simpson R.J. (2010). Exosomes: Extracellular organelles important in intercellular communication. J. Proteom..

[114] Taylor D.D., Gercel-Taylor C. (2008). MicroRNA signatures of tumor-derived exosomes as diagnostic biomarkers of ovarian cancer. Gynecol. Oncol..

[115] Graveel C.R., Calderone H.M., Westerhuis J.J., Winn M.E., Sempere L.F. (2015). Critical analysis of the potential for microRNA biomarkers in breast cancer management. Breast Cancer.

[116] Schwarzenbach H., Hoon D.S., Pantel K. (2014). Cell-free nucleic acids as biomarkers in cancer patients. Nat. Rev. Cancer.

[117] Pritchard C.C., Cheng H.H., Tewari M. (2012). MicroRNA profiling: Approaches and considerations. Nat. Rev. Genet..

[118] Chevillet J.R., Lee I., Briggs H.A., He Y., Wang K. (2014). Issues and prospects of microRNA-based biomarkers in blood and other body fluids. Molecules.

